# The validity of over-the-counter skin, hair, and nail recommendations for adult patients with cancer: A systematic review

**DOI:** 10.1007/s00520-024-08735-5

**Published:** 2024-08-08

**Authors:** Bahar Javdan, Lindsay M. Pattison, Sneha A. Rangu, Emely Tejeda, Beth N. McLellan

**Affiliations:** 1 Rutgers Robert Wood Johnson Medical School, New Brunswick, NJ USA; 2 Present Address: Department of Medicine, Division of Dermatology, Albert Einstein College of Medicine, Bronx, NY USA

**Keywords:** Skincare, Hair, Nails, Chemotherapy, Over the counter recommendations

## Abstract

**Importance:**

Patients undergoing cancer treatment experience a multitude of skin, hair, and nail adverse events, prompting them to use non-evidence-based and often restrictive over-the-counter (OTC) recommendations to alleviate their symptoms. Comprehensively assessing evidence-based OTC modalities is crucial to enable cancer patients to comfortably resume their lives post-treatment and integrate clinically sound practices into their self-care routines.

**Objective:**

Perform a systematic review and assessment of evidence-based OTC skin, hair, and nail care recommendations for adult patients undergoing cancer treatment.

**Evidence review:**

PubMed, Cochrane, Embase, and Medline databases were searched in March 2023 to identify English articles addressing OTC skin, hair, and nail care recommendations for adult patients before, during, and after cancer chemotherapy or radiation therapy (RT). Quality was assessed with Oxford Centre for Evidence Based Medicine criteria.

**Findings:**

2192 unique articles were screened, of which 77 met inclusion criteria consisting of 54 randomized controlled trials (RCT), 8 non-randomized controlled cohorts, 1 non-randomized controlled clinical trial, 3 controlled prospective cohorts, 4 prospective cohorts, 2 controlled clinical trials, 1 prospective comparative study, 2 case reports, and 2 case series discussing 9322 patients. An additional article outside of our database search was included for a total of 78 articles. OTC skin care treatments with the best quality of evidence included moisturizing creams. Our review revealed a paucity of evidence-based hair and nail care practices.

**Conclusions and relevance:**

This systematic review serves to highlight the efficacy of diverse OTC skin, hair, and nail care recommendations for adult cancer patients while encouraging further clinical trials to establish evidence-based management guidelines.

## Introduction

Many patients undergoing cancer therapy experience adverse dermatologic events including radiation dermatitis (RD), alopecia, rashes, hyperpigmentation, hand-foot syndrome (HFS), phototoxicity, and nail dystrophy [1, 2]. These patients often face a multitude of challenges, both physical and emotional, related to the dermatologic effects of their treatments. To help mitigate these issues, patients either independently seek or are encouraged by healthcare providers or peers to follow various skin, hair, and nail care recommendations, many of which are non-evidence based and often restrictive (Table 1). There are frequently no or few citations associated with these recommendations and those that are cited often reference narrative reviews lacking actual evidence from clinical trials. In addition to the questionable validity of these recommendations, the restrictive nature of many can cause undue stress and anxiety for patients.

**Table 1:** Examples of suggested skin, hair, and nail care guidelines

Skin care
Avoid: heat exposure (long and hot showers [3], saunas [4]), shaving the armpit with a straight razor [5], application of perfume, deodorant, powder, and lotion in the treatment site [6], application of greasy creams (e.g. pure petroleum), application of topicals prior to RT [7], wearing clothes made of synthetic material [8], wearing tight clothes[4] Recommend: Shower before each treatment with mild unscented soap [5], wash with mild pH, neutral, or non-alkaline soaps [9], free of fragrances, alcohols, fruit, or plant extracts [4]
Hair care
Avoid: daily shampoo, use of hair clips, barrettes, bobby pins, hair ties, dryers, curling irons, straighteners, hair spray, and dye [10] Recommend: Keep a short hair style [10]
Nail care
Avoid: manicures and pedicures [3], tight shoes [8], prolonged immersion of hands in water, false nails, and acetone remover [4]

It is important to investigate the validity of cancer therapy skin, hair, and nail care recommendations to identify and promote evidence-based practices. By prioritizing evidence-based recommendations, clinicians can offer patients interventions that have been rigorously studied and proven effective, promoting their well-being, and optimizing their quality of life throughout their cancer journey. Moreover, evidence-based practices empower clinicians to make informed decisions tailored to individual patient needs. This systematic review aims to summarize current evidence-based recommendations in the literature as they pertain to skin, hair, and nail care management for adult patients before, during, and after cancer therapy, along with a quality of evidence assessment for each supporting study.

## Methods

We performed a systematic literature search to identify evidence-based OTC skin, hair, and nail care recommendations for adult patients undergoing cancer treatment. Our systematic review follows the Preferred Reporting Items for Systematic Reviews and Meta-analyses (PRISMA) reporting guidelines [11]. Using the PubMed, Cochrane, Embase, and Medline databases, a search for all peer-reviewed articles was performed with the following search terms: “skin care AND chemotherapy,” “skin care AND radiation,” “skin care AND radiotherapy,” “hair care AND chemotherapy,” “hair care AND radiation,” hair care AND radiotherapy,” “nail care AND chemotherapy,” “nail care AND radiation, “nail care AND radiotherapy.”

**Fig. 1:**
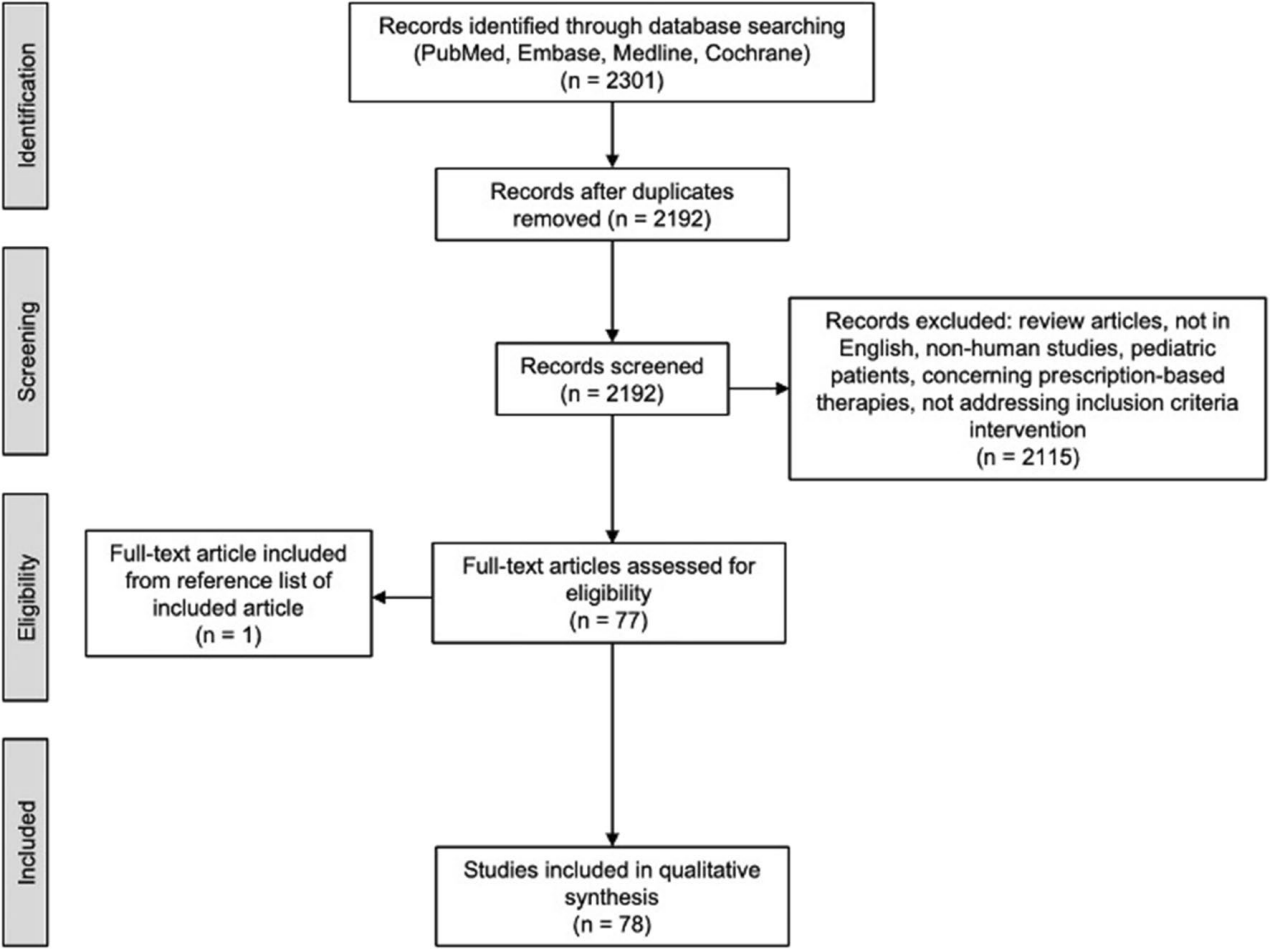
Flowchart illustrating article selection process

The abstracts were independently screened using defined criteria for eligibility. Inclusion criteria specified that papers be: written in English and discuss studies of OTC interventions addressing skin, hair, and nail changes in adults age 19 or older receiving chemotherapy or RT for cancer. References from included reports were reviewed and additional sources that were not initially identified were added. Articles were excluded if they were review articles, not available in full text, not in English, animal studies, or studies of pediatric patients, or involved prescription-based therapies (Fig. 1). Animal studies were omitted because they might not accurately reflect human physiology, treatment response, or adverse effects, thereby limiting their relevance to clinical decision-making for humans. Pediatric studies were excluded because skin, hair, and nail care practices are much more common in adults and differ between adults and children. This leads to varying OTC recommendations influenced by differing physiology, treatment protocols, and potential adverse effects, thereby limiting their direct applicability to the targeted adult cancer patient population and their providers in this review. Four reviewers (B.J., L.M.P, S.A.R., and E.T.) independently screened all titles and abstracts. Articles that met inclusion criteria underwent full-text review. In case of disagreement, a consensus meeting was held to resolve discrepancies. Quality of evidence was used to evaluate the strength of a particular recommendation and was assessed and classified by the Oxford Centre for Evidence-Based Medicine 2011 Levels of Evidence (LoE) as previously described [12]: level 1 (systematic review of RCTs or high-quality randomized controlled trial), level 2 (lesser quality RCT or prospective cohort study), level 3 (case–control study, non-randomized controlled cohort or follow-up study), level 4 (case series), or level 5 (expert opinion, mechanism-based reasoning).

## Results

The initial database search provided 2301 total articles with 2192 unique articles after removal of duplicates. Seventy-seven articles met inclusion criteria consisting of 54 RCTs, 8 non-randomized controlled cohorts, 1 non-randomized controlled clinical trial, 3 controlled prospective cohorts, 4 prospective cohorts, 2 controlled clinical trials, 1 prospective comparative study, 2 case reports, and 2 case series discussing 9322 patients. An additional article, an RCT of 22 patients, that met inclusion criteria was added from a reference list of a screened article. A total of 78 studies, including 77 articles from the database search and an outside search article, were included in our final review. OTC skin care treatments with the best quality of evidence included moisturizing creams and lotions. Treatments with moderate quality of evidence and efficacy included antimicrobials and antiseptics, dressings, and natural products. Our review revealed a paucity of evidence-based hair and nail care practices. Included articles and results are summarized in Table 2.

**Table 2:** Included studies evaluating skin, hair, and nail care treatments in adult patients undergoing cancer therapy

Therapeutic Class	Type of Study	Studied Intervention	Control group Intervention	N	Key findings	Quality of Evidence	Reference
Skin Care							
Basic Hygiene	RCT	Washing was allowed with water and soap	No washing was allowed during RT	99	During RT for breast cancer, washing the irradiated skin was not associated with increased skin toxicity	2	[13]
	RCT	Antiperspirant	Standard care wash	198	There was no statistically significant difference between the skin reaction of the two groups over time	1	[14]
	RCT	Antiperspirant containing aluminum chlorohydrate	Placebo cream	52	As an antiperspirant, topical aluminum chlorohydrate reduced the incidence of grade 2 or 3 palmar-plantar erythrodysesthesia following pegylated liposomal doxorubicin chemotherapy for metastatic breast cancer	2	[15]
Antimicrobials / Antiseptics	RCT	Gentian violet	Nonadherent absorbent	146	The results showed that patients in the two groups did not have any significant difference in wound-healing time, disturbance in mood, sleep, social interaction, appearance, and neck mobility	1	[16]
	RCT	Gentian violet application on wounds	Moist dressing (hydrocolloid)	39	Gentian violet significantly improved wound healing in radiation-induced moist desquamation	2	[17]
	RCT	Aqueous gentian violet vs hydrogel dressing after RT	No treatment	30	The use of hydrogel in moist desquamation provided a shorter wound healing time than control	2	Gollins et al. 2008
	RCT	Chlorhexidine gluconate	Nonmedicated skin cleanser	40	The data suggest that chlorhexidine gluconate does not offer increased protection against perirectal infections in patients undergoing intensive chemotherapy, nor is it more irritating than a nonmedicated skin cleanser	2	[18]
Creams, Ointments, Lotions, and Gels	Controlled prospective cohort	Concomitant treatment with an acidic wash and an acidic emollient (both pH 5.5) (to one forearm)	No treatment (to other forearm)	30	After a 3-week treatment, acidic wash and acidic emollient increase stratum corneum hydration, reduce transepidermal water loss, and increase sebum levels	2	Fluhr et al. 2007
	RCT	Calendula vs Essex cream	No treatment	411	No significant difference in severe acute radiation skin reaction between Calendula cream and aqueous Essex cream	1	Sharp et al. 2013
	Controlled prospective cohort	Biafine cream	No treatment	60	Majority (98%) of patients developed grade 2 or less RD with the use of Biafine cream	2	[19]
	RCT	Biafine	Standard of care	172	Biafine had no overall difference between treatment and control prevention, time to, or duration of RD	1	[20]
	RCT	Natural oil-based emulsion containing allantoin	Aqueous cream	174	Natural oil-based emulsion containing allantoin has similar effects in managing skin toxicity compared with aqueous cream up to week 5,but is less effective at later weeks into radiation treatment and beyond treatment completion (week 6 and beyond)	1	Chan et al. 2014
	RCT	Moisturizing or barrier cream	No treatment	255	Barrier cream significantly reduced the incidence of moist desquamation during RT	1	Laffin et al. 2015
	RCT	Hydrosorb®	Water based spray	270	No significant difference between Hydrosorb® and simple water spray in the treatment of acute RD	1	[21]
	RCT	Aquaphor ointment, Biafine RE cream, or RadiaCareTM gel	Placebo (sterile water mist)	208	None of the products were statistically better than placebo in preventing skin reactions	1	[22]
	RCT	Moisturizing durable barrier cream	10% glycerine cream (sorbolene)	333	The moisturizing durable barrier cream did not reduce the peak skin reaction compared to sorbolene	1	Graham et al. 2013
	Controlled prospective cohort	Creme or powder	No treatment	12	No relevant difference in favor of care with creme or powder according to objective as well as subjective assessment criteria	2	Schreck et al. 2002
	Non-randomized controlled clinical trial	Topical phytotherapic product (Capilen® cream)	No medication	30	Capilen® cream showed a significant delay in onset of acute dermatitis	3	Stefanelli et al. 2014
	RCT	Prophylactic trolamine emulsion vs interventional trolamine emulsion	Declared institutional preference	547	No advantage for the use of trolamine in reducing the incidence of grade 2 or higher RD or improving patient-reported quality of life	1	[23]
	Non-randomized controlled cohort	Skin moisturizers with sunscreen and lymecycline 300 mg/daily	No treatment	51	At 3-month evaluation, 27.4% patients had grade 2 skin toxicity, of which 71.4% showed toxicities during the 1st month; 21.4%, in the 2nd month; and 7.2%, in the 3rd month	3	Grande et al. 2013
	RCT	Essential oil	Standard of care	24	The essential oil mixture did not provide a better skin protectant effect than standard of care	2	Halm et al. 2014
	RCT	Hyaluronic acid based gel (RadiaPlex)	Aquaphor	74	Hyaluronic acid gel had a significantly higher rate of RD and worse dermatitis in the breast area compared to Aquaphor treatment	1	Pinnix et al. 2012
	RCT	Hyaluronan formulation on breast undergoing RT	Control cream (placebo)	28	No significant difference in acute skin toxicity between hyaluronan formulation cream and placebo cream	2	Rahimi et al. 2019
	RCT	Topical cream with lipid nanoparticles containing 2% vitamin E, 3 × per day after RT sessions and up 2 weeks after RT cessation	Control 1: cream without lipid nanoparticles or Vitamin E Control 2: cream with empty lipid nanoparticles and no vitamin E	40	Vitamin E-containing cream had a delayed onset of RD compared to control group, but no difference in grades of RD	1	[24]
	RCT	Trolamine emulsion for RT for head and neck squamous cell carcinoma	Standard of care	30	Trolamine emulsion significantly reduced the grade of RD compared to standard of care	2	Abbas et al. 2008
	RCT	Verum ointment Mapisal (antioxidant containing ointment)	Placebo ointment	32	Zero patients in the Mapisal ointment group developed palmoplantar erythrodysesthesia after treatment with Caelyx compared to 71% in the placebo group	1	Jung et al. 2017
	RCT	Treatment with RayGel	Placebo treatment	30	The group receiving RayGel had a lower average score of percent of breast skin involved and grade of reaction versus the placebo group	1	Miko Enomoto et al. 2005
	RCT	New antioxidant ointment	10% urea cream	152	10% urea cream onset to any-grade HFS was significantly longer compared to new antioxidant ointment	1	[25]
	RCT	Human recombinant EGF-based cream	General supportive skin care	40	EGF based cream had a significantly lower incidence of grade 3 RD compared to the control group	1	Kong et al. 2013
	Non-randomized controlled cohort	Epidermal growth factor (EGF) ointment	No treatment	51	The EGF ointment significantly improved rash/acne and itching	3	Hwang et al. 2016
	RCT	Sucralfate or aqueous cream	No cream	357	No differences were found in the severity of skin reactions or levels of discomfort between the two groups	1	Wells et al. 2004
	RCT	Sucralfate	Placebo	50	Acute radiation reaction of the skin was statistically significantly prevented by the sucralfate cream and prompted faster recovery of the skin compared to placebo	2	Maiche et al. 1994
	Non-randomized controlled cohort	Sucralfate lotion	No treatment	21	The sucralfate-containing lotion did not provide systematic RD prevention	3	Falkowski et al. 2011
	RCT	Urea cream	Placebo	288	Urea cream had a lower incidence of severe HFS at 2 weeks and reduced the tendency of HFS development	1	Lee et al. 2020
	Prospective cohort study	Treatment with lotion containing 3% urea, polidocanol and hyaluronic acid	No treatment	98	Percentage of patients who did not develop RD was significantly higher in the treatment group, as well as lower incidence of RD, lower grade of toxicity, and lower proportion of RD grade 2 or higher	2	[26]
	RCT	Urea cream	Supportive cream	871	Urea cream prophylaxis reduced HFS rates, extended the time to first occurrence of HFS, and improved patient quality of life compared with control group	1	Ren et al. 2015
Dressings	RCT	Mepitel Film	Biafine cream	44	Skin reaction severity (combined RISRAS score) underneath Mepitel Film was decreased by 30% (P < 0.001) and moist desquamation rates by 41% (P < 0.001)	1	[27]
	Non-randomized controlled cohort	Silver-leaf nylon dressing (SLND)	Standard skin care	30	The results of this study suggest that SLND is effective in reducing RD	3	[28]
	RCT	Silver clear nylon dressing	Standard skin care	42	Silver clear nylon dressing is effective in reducing RD in patients with lower gastrointestinal cancer treated with combined chemotherapy and radiation treatment	1	[29]
	Prospective cohort study	Polymeric membrane dressing (PolyMem®)	Standard treatment (topical aqueous cream at the start of RT with the addition of paraffin gauze when moist desquamation occurred)	20	PolyMem reduced pain and inflammation, improved sleep patterns, improved healing rates, and improved quality of life	2	[30]
	RCT	3 M Cavilon No-Sting Barrier Film	Sorbolene cream (with 10% glycerin)	61	No-Sting reduces the duration and frequency of radiation-induced moist desquamation	1	[31]
	Controlled clinical trial	Airwall film dressing (Group A)	Standard skin management (group B)	271	Film dressing using Airwall reduced the severity of acute RD without delaying the response time of the skin to proton beam irradiation compared with standard skin management	3	[32]
	RCT	Hydrofilm polyurethan film dressing	Urea 5% lotion	62	In the Hydrofilm compartments, mean maximum RTOG/EORTC RD severity grades were significantly reduced and photospectrometric measurements showed significantly reduced erythema severity, as compared to the control compartments. Hydrofilm completely prevented moist desquamation and significantly reduced patients' subjective experience of itching and pain	2	[33]
	RCT	StrataXRT® (silicone-based film-forming gel dressing)	Sorbolene (usual care)	197	The StrataXRT® group had lower incidence of grade 2 and grade 3 skin toxicity compared to control. StrataXRT® was associated with reduced risks of developing grade 2 and 3 skin toxicity throughout treatment compared to the control group	1	[34]
	RCT	Mepilex lite dressings	Standard of care	88	RD in the treatment group healed in a median of 16 days, vs 23 days in control group	1	[35]
	RCT	Hydrogel use during moist desquamation post RT	Tricotex (dry dressing)	357	Participants who used the hydrogel dressing after moist desquamation had a significantly longer time to heal vs those who used the simple dry dressing, Tricotex	1	[36]
Natural Products	RCT	Powder or aloe cream	Placebo cream	248	Aloe was not associated with acute skin toxicity or symptom severity. Compared to the dry powder regimen, both study creams were associated increased skin reaction toxicity	1	[37]
	RCT	Topical aloe vera gel	Topical aqueous cream	225	Aloe vera gel did not significantly reduce radiation-induced skin side effects. Aqueous cream reduced radiation-induced dry desquamation and pain	1	[38]
	RCT	Mild soap with aloe vera gel	Mild soap alone	73	When added to the soap regimen, aloe demonstrated a protective effect on skin with increasing cumulative radiation dose (> 2700 cGy)	2	[39]
	RCT	Curcumin vs HPR plus	Placebo	191	Although no significant effects were observed, prophylactic treatment with topical curcumin may minimize skin reactions and pain for patients with high breast separation (≥ 25 cm)	1	[40]
	RCT	Formulation A (capparis spinosa, opuntia coccinellifera, olive leaf extracts); formulation B (Biafine, nonsteroid topical)	No treatment	68	No overall difference between treatment and control prevention, time to, or duration of RD	2	Rizza et al. 2010
	RCT	Topical Calendula officinalis (Calendula)	Sorbolene (standard of care)	81	No significant difference was observed between Calendula and standard of care (Sorbolene) for the prevention of RD	1	[41]
	RCT	Epigallocatechin-3-gallate (EGCG) solution	Placebo (saline)	165	The treatment group was significantly associated with lower grade 2 RD than the control group (p = 0.008)	1	[42]
	Prospective comparative study	Silymarin-based cream (Leviaderm) in preventing RD	Standard of care	101	Compared to placebo cream, prophylactic Silymarin-based cream Leviaderm(®) use was associated with significant prolongation of the onset of RD and fewer skin reactions	2	Becker-Schiebe et al. 2011
	RCT	Homeopathic belladonna 7cH, X-ray 15cH in the treatment of acute RD	Placebo	66	There was no statistical difference between the treatment and control groups for breast skin color, warmth, swelling, and pigmentation during RT	2	Balzarini et al. 2000
	Case series	Leptospermum honey	None	4	Application of leptospermum honey was associated with improvement in size and condition of wound/periwound area and a reduction in pain and complete healing was noted in 2.5 weeks (with honey and paraffin) and 6 weeks (with honey-soaked hydrofiber rope)	4	Robson et al. 2009
Other Skin Care	RCT	Specific hydrotherapy	Supportive care	68	Most subjects showed significantly greater improvement in quality of life, reduction of side effects, body image, and healing	1	Dalenc et al. 2018
	RCT	Photobiomodulation therapy	Placebo	120	Photobiomodulation therapy is effective in reducing the incidence of moist desquamation in breast cancer patients undergoing RT	1	Robijns et al. 2019
	RCT	Customized compression garments	Observation	56	Lower limb lymphedema incidence was reduced in the compression garment group compared to the control group, but not statistically significant	2	Hnin et al. 2018
	RCT	Addition of manual lymphatic drainage	Standard therapy consisted of use of a compression garment, exercises and information about lymphedema and skin care	42	The study showed that both groups obtained a significant reduction in edema and that manual lymphatic drainage did not contribute significantly to reduce edema volume	1	Andersen et al. 2000
	Case series	Cavilon Advanced Skin Protectant	None	4	Cavilon Advanced Skin Protectant has beneficial effects and is safe to use in the management of acute RD in patients with cancer of different etiologies	4	Robijns et al. 2022
	Case report	Lactoline	None	1	Lactoline was feasible and safe, and the patient developed only a slight case of RD	4	Häfner et al. 2013
	Case report	Kinesio tape	None	1	Kinesio tape had a significant effect on the reduction of lymphedema and accelerates healing effects compared to standard methods	4	Taradaj et al. 2014
Vitamin K	Controlled clinical trial	Vitamin K1 cream	No treatment	40	Prophylactic topical vitamin K1 cream significantly reduced the development of acneiform rash in patients with metastatic colorectal cancer treated with cetuximab	3	Jo Jet al. 2013
	Prospective cohort study	Vitamin K1 cream	No treatment	41	Vitamin K1 was associated with a lower proportion of grade 2 rash (25%) and grade 3 rash in patients with metastatic colorectal cancer treated with cetuximab	2	[43]
	RCT	Vitamin K3 cream for cetuximab induced rash prophylactically or during papulopustular eruptions	Placebo cream	30	Compared to the control group, vitamin K3 cream did not significantly reduce papulopustular eruptions or EGFR and pEGFR markers on immunohistochemical stain	2	[44]
Hair Care							
	RCT	Maintain normal hair washing	No hair washing	109	The practice of normal hair washing is not associated with increased severity of adverse skin reaction	1	[45]
	RCT	Topical lotion (Batch DT023) is a botanical drug under development containing a novel patented blend of 4 botanical ingredients: citrus, cocoa, guarana, and onion	Placebo	35	After 6 months, hair density and thickness had increased compared with baseline	2	[46]
	RCT	2% minoxidil topical solution	Placebo	22	Minoxidil significantly decreased the duration of alopecia post-chemotherapy	1	[47]
Scalp Cooling	Prospective cohort study	SC	No treatment	266	SC was effective in preventing chemotherapy-induced hair loss in 52% of patients	3	[48]
	Non-randomized controlled cohort	SC	No treatment	74	In anthracycline-treated patients, total prevention of hair loss was observed, whereas hair loss in paclitaxel/docetaxel-treated patients was minimal to none	3	[49]
	Non-randomized controlled cohort	Cold cap (CC) vs Paxman psc-2 machine (PAX)	No cooling	238	Overall, cooling (PAX and CC combined) reduced risk of alopecia by 78%. CC and PAX prophylaxis led to the same degree of prevention of alopecia	3	[50]
	Non-randomized controlled cohort	SC	No SC	53	81% of scalp-cooled patients did not require head covering versus 27% of non-scalp-cooled patients	3	[51]
	RCT	SC 20 min	SC 45 min	134	No significant difference between 2 groups	1	Komen et al. 2016
	RCT	SC 150 min	SC 90 min	102	Longer duration of SC did not significantly decrease the need for head covering. However, grades 2–3 alopecia was seen less often with prolonged post-infusion SC	1	[52]
Nail Care							
Nail Cryotherapy	RCT	Frozen gel gloves 15 min prior to infusion, 1 h during the infusion, and 15 min post-infusion	Not wearing glove	21	No significant differences in nail and skin toxicity were observed between the gloved and non-gloved hands	2	[53]
	Non-randomized controlled cohort	Frozen glove 15 min prior to infusion, 1 h during infusion, and 15 min post infusion	Left hand left unprotected as control	45	Skin and nail toxicity were significantly lower in the gloved hand compared with the control hand (P = 0.0001)	2	[54]
	RCT	Nail covering (painting nails with dark nail varnish) and OnicoLife® treatment	Standard care (lifestyle and hand hygiene to prevent nail infection and damage)	105	Compared to patients using nail polish, those receiving specialist drops or standard care experienced less nail toxicity	1	[55]

## Basic hygiene and routine care

​​Recommendations pertaining to basic hygiene and routine care exist in other reviews in literature that were not supported by any studies in our systematic review including: avoidance of manicures and pedicures [3], keeping a short hair style, avoiding daily shampoo, avoiding hair manipulation such as using hair clips, dryers, curling irons, dye [10], avoiding shaving the armpit with a straight razor [5], and avoidance of perfume, deodorant, powder, and lotion in the treatment site [6].

We found several evidence-based studies that contraindicate the previous suggestions. In a study, washing the skin with soap and water during the course of treatment was not associated with increased skin toxicity [13]. A study evaluating aluminum-based antiperspirant use in women receiving external beam RT for breast cancer found that antiperspirant use was not associated with any significant skin reaction compared to the control group [14]. Interestingly, in another study, aluminum-based antiperspirant use in breast cancer patients treated with pegylated liposomal doxorubicin was associated with a decreased incidence of grade 2 or 3 palmar-plantar erythrodysesthesia [15].

## Skin care

### Creams, ointments, lotions, and gels

Dermatitis, itchiness, xerosis, and erythema are common side effects of RT but can be ameliorated with the use of creams, ointments, lotions, and/or gels [1, 2]. Results from a large multi-institutional study found that thin or moderately applied topical agents have minimal effect on RT skin dose [56], negating suggestions that topicals should be avoided prior to RT. Application of topical vitamin E, RayGel, phytotherapic, urea, or antioxidant creams reduced onset and severity of RD [24–26, 57]. Urea-containing creams have shown benefit in preventing HFS during and following chemotherapy [25, 26]. Analgesic-containing gels, such as trolamine, may also play a role in alleviating acute RD by promoting wound healing. Trolamine use is associated with conflicting results, warranting additional studies of analgesic use for RD [23, 58].

Several published studies have found no benefits of certain emollients in acute skin reactions. Hydrosorb and Radiacare gel have been found to be ineffective in treating RD, while Biafine cream has demonstrated mixed results [19–22]. It is important for clinicians to counsel patients undergoing RT on the use of ineffective creams and gels, which may be found and ordered online.

### Dressings

Dressings are often used to treat wounds from RT and since skin toxicity from RT can result in desquamation, many studies have evaluated the utility of dressings in the prevention and treatment of RD. Studies show that Mepitel film, silver nylon dressings, polymeric membrane dressings, 3 M Cavilon No-String Barrier film, Airwall film, Polyurethane hydrofilm, StrataXRT® silicone film, and Mepilex Lite dressing are effective at reducing the duration and frequency of RD [27–35]. One study found that a wet dressing, Hydrogel, resulted in a significant increase in healing time compared to a dry dressing, Tricotex [36].

### Vitamin K

Epidermal growth factor receptor (EGFR) inhibitors such as cetuximab are associated with skin toxicity, namely, papulopustular (acneiform) eruptions following chemotherapy [59]. While there are currently no standard OTC available treatments in preventing EGFR inhibitor induced acneiform rash, vitamin K has been studied as a possible intervention [60].

Studies have found mixed results with the use of vitamin K cream, with a few studies reporting no reduction in the number of cetuximab induced papulopustular eruptions after use of vitamin K1 and vitamin K3 [44, 61]. However, there have been a few reports of lower proportions of grade 2 and grade 3 rash after use of vitamin K cream [43, 62]. Hofheinz et al. found that combination therapy did not decrease grade 2 + skin rash. There is currently no evidence-based recommendation to use vitamin K to prevent EGFR induced skin toxicity [60].

### Natural products

Naturally-derived compounds are often incorporated into skincare regimens for their potential anti-inflammatory and antioxidant benefits for RD [39]. Three studies in this review evaluated the efficacy of aloe in reducing adverse skin reactions in patients undergoing RT. In two studies, aloe did not significantly reduce RD compared to either placebo or topical aqueous cream [37, 38]. In another study, adding aloe to a mild soap skin washing regimen demonstrated a protective effect as the cumulative radiation dose increased over time [63].

Other naturally derived compounds that have been investigated in trials include curcumin, Calendula, and Epigallocatechin-3-gallate (EGCG), a bioactive constituent of green tea, all of which demonstrate antioxidant properties [64–66]. Wolf et al. found that prophylactic treatment with topical curcumin was effective in minimizing skin reactions and pain for patients with high breast separation (i.e., larger breast size) at the end of RT [40]. In a RCT comparing topical Calendula cream versus standard of care (Sorbolene), no significant difference was observed for the prevention of RD [41]. Zhao et al. investigated whether EGCG can reduce the incidence of RD in patients after breast cancer surgery and found that EGCG prophylaxis significantly reduced both the incidence and severity of RD [42]. Cumulatively, these studies suggest that naturally-derived compounds with potential antioxidant properties may confer a protective effect against oxidative stress induced by free radicals during radiation treatment.

### Antimicrobials and antiseptics

There are very few studies looking at non-prescription based antimicrobials or antiseptics, such as Gentian violet and chlorhexidine. Gentian violet has shown mixed results in treating RD [16, 17]. Chlorhexidine did not confer a protective benefit against infections in patients undergoing chemotherapy [18].

### Hair care

Scalp cooling (SC) was the most commonly used hair care practice investigated in our review for chemotherapy-induced alopecia. In several trials, SC demonstrated efficacy in preventing chemotherapy-induced hair loss and may work better for patients receiving certain chemotherapies such as anthracyclines [48–51]. Additionally, prolonged post-infusion SC has been associated with better outcomes [52].

A few other studies investigated techniques such as the use of topicals and hair washing practices to prevent hair loss during chemotherapy. A study in patients with alopecia secondary to chemotherapy demonstrated that topical 2% minoxidil significantly reduced the period of baldness [47]. A novel topical containing a blend of four botanical ingredients (citrus, cocoa, guarana, and onion) was shown to increase hair density and thickness compared to baseline after 6 months of use [46]. Various hair washing techniques have not demonstrated any significant difference in hair loss compared to control [45]. Although avoidance of hair dye is a common recommendation, we did not find any articles that referenced hair dye or other chemicals.

### Nail care

Nail toxicity has been a well-documented complication of chemotherapy, particularly that of taxane use, causing both functional impairment and psychological distress [67, 68]. Two studies investigated the use of cryotherapy to prevent docetaxel-induced hand and nail toxicity with conflicting results. While one trial with 41 patients found that onycholysis and skin toxicity were significantly reduced in the frozen glove protected hand [54], another trial with 21 patients found no significant difference between cutaneous hand toxicity in the gloved and non-gloved hands [53]. Further studies are needed to investigate whether cryotherapy can be used as an effective intervention to reduce taxane-induced nail and skin toxicity.

Morrison et al. investigated 2 interventions compared to standard of care for taxane-induced nail toxicity in women with early breast cancer [55]. Standard of care included lifestyle and hand hygiene practices aimed to prevent nail infection and damage including wearing household gloves when using chemicals, nail filing rather than cutting, and moisturizing hands around the fingernails. Two interventions included nail coverings (painting nails with dark nail varnish thought to prevent UV-induced damage) and Onicolife, a nail-specific medical advice consisting of anti-inflammatory and antiseptic compounds to protect tender and fragile nails). Compared to the use of dark nail varnish, standard care and the specialized Onicolife nail drops and nail oil were significantly associated with less nail toxicity [55].

## Discussion

Patients undergoing cancer treatment often experience significant psychological distress and physical discomfort due to changes in their skin, hair, and nails. Unsubstantiated recommendations can add to this distress and hinder patients from resuming their normal lives during and post-cancer therapy, leading to unnecessary stress and anxiety. Clinicians play a pivotal role in assisting patients in maintaining their quality of life and sense of identity throughout the entire cancer treatment process. Therefore, it is imperative for clinicians to identify and counsel patients on evidence-based, effective, safe, and tolerable options for preventing and treating dermatologic disorders associated with cancer treatments. Additionally, it is important to individualize recommendations based on patient values and available evidence.

This systematic review underscores the efficacy of various OTC treatment modalities, with moisturizing creams and lotions having the highest quality of evidence and efficacy. Treatments with moderate quality of evidence and efficacy included antimicrobials and antiseptics, dressings, and natural products. It is important to acknowledge the varying quality of evidence for scalp cooling, a commonly used practice for chemotherapy-induced alopecia, necessitating larger clinical trials for a more comprehensive understanding of its efficacy and safety. Contradictory findings on the use of cryotherapy in taxane-induced nail toxicity as well as nail polishes and nail drops warrant further research in nail care practices. Importantly, recommendations on basic hygiene and routine care, such as avoiding certain practices, lacked support in the systematic review.

The results presented here must be interpreted with caution due to the variability in study sizes and the quality of study design. Certain treatment modalities were characterized by conflicting results and may not be generalizable to all patients. Different studies demonstrate short-term versus long-term benefits, emphasizing the importance of considering the duration of improvement for a given therapy. While the majority of studies in our review involved breast cancer patients, it is important to investigate evidence-based treatments in other cancer types owing to differences in the skin of various body regions. Compared to trials investigating evidence-based skin care regimens, there is a paucity of trials investigating those of hair and nail care.

In this systematic review, we summarized current evidence-based OTC recommendations in the literature as they pertain to skin, hair, and nail care management for adult patients before, during, and after cancer therapy, along with a quality of evidence assessment for each study. We hope that this review serves as a comprehensive guide for clinicians and patients to incorporate evidence-based recommendations and inspires further clinical trials to advance the field of supportive oncodermatology. This approach not only enhances quality of care but also fosters a sense of trust between healthcare providers and patients.

## Data Availability

All included studies and their respective data sources are referenced within the manuscript.

## Abbreviations

*OTC*: Over the counter
*RD*: Radiation dermatitis
*RT*: Radiation therapy
*HFS*: Hand foot syndrome
*PRISMA*: Preferred reporting items for systematic reviews and meta-analyses
*LoE*: Level of evidence
*RCT*: Randomized controlled trialEGFR—epidermal growth factor receptor
*EGCG*: Epigallocatechin-3-gallate
*SC*: Scalp cooling
